# Immersive virtual reality in a northern Queensland haemodialysis unit: Study protocol for a cross-over randomized controlled feasibility trial (ACTRN12621000732886)

**DOI:** 10.1016/j.conctc.2022.100956

**Published:** 2022-06-30

**Authors:** Wendy Smyth, Joleen McArdle, John Body-Dempsey, Valli Manickam, Anne Swinbourne, Ickjai Lee, Jason Holdsworth, Olumuyiwa Omonaiye, Cate Nagle

**Affiliations:** a Townsville Hospital and Health Service, PO Box 670, Townsville, Queensland, 4810, Australia; b College of Healthcare Sciences, James Cook University, Townsville, Queensland, 4811, Australia; c College of Science & Engineering, James Cook University, Cairns, Queensland, 4878, Australia

## Abstract

**Introduction:**

Despite being a life-preserving medical treatment, the demands of haemodialysis are a significant impost on individuals, posing considerable burdens on their work, vocational activities and involvement with family and community. In our region, patients who have had to relocate considerable distances to a regional city for dialysis, and First Nations people, are less likely to attend all scheduled dialysis sessions. Virtual reality (VR) has been shown to improve engagement with care of people on haemodialysis.

This manuscript describes the protocol for a cross-over randomised controlled trial (RCT) that will explore the impact of an immersive VR experience for patients attending a northern Queensland, Australia, haemodialysis service.

**Methods:**

The design is a crossover RCT, with 8 clusters according to haemodialysis location and schedule. Clusters (5 participants in each) will be randomized by computer program. Participants in the trial will be patients who undergo haemodialysis three times/week at one of two dialysis units. During the 4-week intervention period (12 haemodialysis sessions), participants will be provided a headset with vision representative of the natural environment, and with audio. The 4-week control period will comprise usual activities, such as watching television, reading and sleeping. Outcomes will be measured by participants': attendance at scheduled dialysis sessions; adherence to lifestyle modifications; wellbeing, anxiety and depression; acceptability and usability of VR; and adverse events such as nausea. The feasibility and acceptability of the intervention from clinicians’ perspectives will also be explored.

**Discussion:**

If this VR intervention is feasible, then participants may engage more with haemodialysis regimens and self-care in this very clinical environment.

**Trial registration:**

ACTRN12621000732886.

## 1 Introduction

Despite being a life-preserving medical treatment, the demands of haemodialysis place a significant impost on individuals, with substantial burdens on work, vocational activities and involvement with family [1], often leading to poor attendance at clinic appointments and poor adherence to treatment plans [2]. Factors such as geographical accessibility and cultural acceptability of treatment procedures have been associated with irregular clinic attendance among patients on long-term haemodialysis [3,4]. From a previous retrospective chart review [2], it is known that Aboriginal and/or Torres Strait Islander patients were statistically less likely to attend all scheduled dialysis session, as were patients who have had to relocate from rural settings to the regional city for their dialysis. Interventions to optimise engagement with care and avoid emergent dialysis are urgently needed.

The provision of such interventions is particularly important for First Nation peoples. In northern Queensland the prevalence of end-stage kidney disease demonstrates an inequity: approximately 65% of the Townsville Hospital and Health Service renal patients are of Aboriginal and/or Torres Strait Islander descent, whilst this group constitutes only 7.9% of the Health Service's residents [5].

Findings from our systematic review investigating the effects of virtual reality (VR) on the lives of people receiving haemodialysis [6] favour the use of VR to improve engagement with treatment and thus physical health. However, no studies have been undertaken in Australia with individuals undergoing haemodialysis.

### **1.1** Background

Immersive VR, where a person is exposed to video and sound via a head-mounted display and is isolated from the external world [7], has been shown to be more effective across different domains and settings than traditional two-dimensional (2D) or three-dimensional (3D) video. For example, participants reported greater ‘presence’ when playing a video game in immersive VR format compared to 2D or 3D formats [8], and a greater sense of awe [9,10], and better cognitive function (memory) [11]. Findings from randomised controlled trials (RCTs) of VR in haemodialysis sessions have produced different responses with respect to depression. Non-immersive virtual reality combined with exercise significantly reduced depression in a sample of patients undergoing haemodialysis in Qatar [12] but had no effect in a similar study undertaken in Brazil [13]; different measurement tools were used [6].

It is acknowledged that VR may have the potential to evoke nausea or a sense of motion sickness, as identified by a crossover observational study involving volunteers watching 3D movies [14]. However, a quasi-experimental study undertaken in a haemodialysis setting [15] demonstrated that immersive VR did not worsen a participant's usual symptoms of headaches or nausea. A recent systematic review identified that VR was generally acceptable and safe in a haemodialysis setting, although most studies have used non-immersive VR to promote exercise [6].

Haemodialysis units are clinical, medicalised environments. It is proposed that a VR intervention that is engaging, such as the opportunity to ‘travel through landscapes’, will enable a person to feel more at ease and disengaged from the aspects of the surrounding treatment environment which may arouse anxiety.

The aims of this study are to explore the feasibility of an immersive VR experience for patients undergoing haemodialysis; and to inform a multi-centred RCT on the effects of VR for patients undergoing haemodialysis.

## 2 Methods

### 2.1 Study objectives

The primary objective is to:

- compare participants' adherence to haemodialysis regimens (with respect to attendance at scheduled dialysis sessions) between the intervention and control phase.

The secondary objectives are to:

- compare participants' adherence to haemodialysis regimens (with respect to adherence to fluid allowances) between the intervention and control phases;
- measure change in ratings of quality of life, engagement with self-care and other psychological measures reported by participants between the intervention and control phases;
- measure usage and usability of VR by participants;
- assess the acceptability and appropriateness of using VR during haemodialysis from the perspectives of patients; and
- assess the acceptability and appropriateness of using VR during haemodialysis from the perspectives of clinical staff.

### 2.2 Project design

A crossover RCT will be conducted where participants are their own control. Such a study design requires fewer participants, maximises equivalence between groups and controls for confounding variables [16], compared to traditional parallel RCTs. Consistent with this design, a one-week washout period between the intervention and control phases will be included to minimise any carry over effects [16]. Clusters have been determined by place of dialysis (in-centre or satellite dialysis unit), and scheduled days of dialysis. Each of the 8 clusters will consist of 5 participants (as described later in section 2.9.4).

### 2.3 Intervention

An immersive VR experience (choice of simulations of 30–40 min in duration) can be used at any time during each dialysis session. All-in-one Oculus Quest 2 headsets, not requiring an external computer to connect, will be used in this study to provide the best immersive experience and performance possible. Oculus Quest 2 devices are equipped with 64 Gb, two 6 Degree-of-Freedom controllers to support both orientation and positional tracking and can provide high resolution with lower latency to maximise virtual experience and minimise motion sickness [17]. Several VR simulations will be created using localised natural sounds and scenes to have more contextualised immersive experiences. Headsets will be distributed to each participant for the period of the intervention; they will be cleaned as per the Health Service's infection prevention and control practices between participant groups (see safety considerations/Patient safety section). Headsets have been fitted with spacers to accommodate participants wearing their glasses if they wish. The Intervention period will be four weeks' duration.

### 2.4 Control

The Control period will be four weeks’ duration and involve usual activities, such as watching television, sleeping and reading.

### 2.5 Project setting and population

The project is set in the in-centre dialysis unit at Townsville University Hospital (TUH) and the satellite dialysis unit at North Ward (NW). There are approximately 110 patients undergoing haemodialysis at TUH and 44 patients at NW.

### 2.6 Project duration/timeline

This study is anticipated to take two years. If additional equipment is available, it may be possible to complete the crossovers earlier than anticipated.

### 2.7 Participants

There are two groups of participants: one group comprises patients undergoing haemodialysis, and another group comprises clinicians caring for patients undergoing haemodialysis. Target users are a small group of patients undergoing haemodialysis who will provide feedback to assist the development of the VR experience video.

#### 2.7.1 Group 1: Patients undergoing haemodialysis

Selection criteria are informed by a previous study [18]. Outpatients attending haemodialysis at TUH or NW with the following characteristics will be included: 18 years or older; in treatment with three haemodialysis sessions per week; haemodialysis duration of 3 h or greater; no history of severe migraines; and orientated to time and space. It is expected that some participants will be First Nations people.

#### 2.7.2 Group 2: Clinicians

Clinicians (nurses, doctors and health workers) providing care to patients in Participant Group 1 during the VR intervention.

### 2.8 Sample size calculation

The sample size of Group 1 (patients) of n = 40 is informed by other feasibility studies included in our recent systematic review [6]. The within-subject crossover design minimises sample variability as all participants act as their own controls [19]. Given that the current study is a feasibility analysis, there is less emphasis on hypothesis testing and more upon the appropriateness of the intervention and applicability of the measures for assessing physical and psychological status in this patient population. Our sample size for this feasibility study is based upon empirical literature reports of similar studies and practical limitations upon recruitment, rather than statistical calculations.

With regards to the clinicians (Group 2), we will invite all clinicians (nurses, medical officers, allied health staff) to complete an anonymous questionnaire. It is anticipated that approximately 50% of clinicians will participate.

### 2.9 Project outcomes

The primary outcome is attendance at scheduled dialysis sessions.

The secondary outcomes are:

- Adherence to fluid allowances between dialysis sessions;
- Health related quality of life;
- Engagement with self-care;
- Depression and anxiety;
- Usage, acceptability and usability of VR; and
- Participants' (patients and clinicians) satisfaction with VR.

#### 2.9.1 Project outcome measures

Note that the crossover design means that differences being examined are *within* participants [16]. That is, outcomes in the intervention phase are compared to outcomes in the control phase for all participants.

##### 2.9.1.1 Adherence to haemodialysis regimens will be measured by

- difference in participants' attendance rates when in the intervention phase compared to the control (non-intervention) phase; and
- difference in proportions of participants' adherence to fluid allowances, defined as gaining, on average, no more than 1 kg per day between dialysis sessions in the intervention phase compared to the control phase [2].

##### 2.9.1.2 Health-related quality of life and engagement with self-care

This will be assessed by the use of the Australian Assessment of Quality of Life (AQoL) scale (6D) [20]. The scale consists of 20 items across 6 dimensions (independent living, relationships, mental health, coping, pain, and senses), and participants are asked which of five descriptive statements best describes their current experience in terms of the item of interest. The scale was chosen for its brevity, validity and conversational style [20]. Reliability of the tool (Cronbach's α) has been found to range between 0.73 and 0.84 [21]. An example of one of the questions in the scale asks the participant to describe their situation over the past week, with respect to “How much energy do you have to do the things you want to do”.

##### 2.9.1.3 Depression and anxiety

This will be assessed by the amended Kessler Psychological Distress Scale – 5 (K-5), which contains 5 statements about an individual's psychological state in the previous 4 weeks [22]. Respondents are asked the extent to which they have felt each of these states (feeling nervous, without hope, restless or jumpy, that everything was an effort, and sad). The K-5 was chosen as it is extensively normed in non-Indigenous populations and it forms part of the Social and Emotional Wellbeing module for Aboriginal and Torres Strait Islander Peoples used by the Australian Institute of Health and Welfare [22]. A recent study assessing the psychometric properties of the K-5 with a sample of Aboriginal and Torres Strait Islanders demonstrated good internal consistency/reliability (α = 0.89), and the scale was deemed to have clinical utility as a screening tool for depression and anxiety with the score of ≥11/25 [23].

##### 2.9.1.4 Participants' satisfaction, usage and usability, and clinicians’ satisfaction measurements

Participants' satisfaction with, and immersion in, the VR experience will be measured with the User Engagement Scale – Short Form [24], modified and incorporated into a conversational interview, congruent with a ‘yarning’ method of Indigenous research [25]. Usage and usability of the VR will be measured by the percentage of dialysis occasions that patients use the VR headset, the amount of time within a session the participant used the headset, whether VR is used at the beginning, middle or towards the end of the dialysis sessions. These data are gathered by the headset and uploaded automatically from it. Any difficulties encountered in its use will be likely reported in the delivery of the User Engagement scale above.

Clinicians’ satisfaction will be measured by a questionnaire asking about the acceptability, feasibility, benefits and barriers to using VR in a haemodialysis setting. Due to the novelty of VR in treatment environments, no appropriate standardised assessments for this population group using this technique currently exist. Clinical staff will be asked to complete a survey adapted from Lindner et al. [26]. This assessment asks about clinician familiarity with the medium both in and outside the clinical setting. Respondents are presented with positive and negative aspects of the use of VR (e.g. patient mood improvement versus cost of technology) and asked how concerned they are about each issue. There is an option at the end of the questionnaire for open-ended answers for additional comments.

#### 2.9.2 Recruitment and consent of participants

Flyers will be displayed in the TUH in-centre and NW satellite renal units, alerting staff and patients to the study. The study will be explained to the staff of the units during meetings and usual communication channels. Training about the recruitment process will be provided to staff who have never been involved in this process if required.

##### 2.9.2.1 Recruitment of patient participants

A significant proportion of patients undergoing haemodialysis are Aboriginal and/or Torres Strait Islanders, thus it is conceivable that many of the participants will also be Aboriginal and/or Torres Strait Islanders. The wording of the participant information sheet and the design of the study has been guided by the Clinical Nurse Consultant – Indigenous at TUH and the health service's Aboriginal and Torres Strait Islander Health Leadership Advisory Council (ATSIHLAC). Information about the study, including potential benefits such as feeling more relaxed, and potential risks such as motion sickness, will be provided to all potential participants and adequate time will be provided to answer questions about participation and consider whether they wish to participate. Written consent will be obtained.

Invitations to participate will commence at least two weeks before the start of the intervention. The male Senior Health Worker (PI – JB-D) and the female Health Worker (RD) in the renal unit will be instrumental in the recruitment process. They will discuss the option of participating during their routine care of eligible patients. Other nurses in the Renal Unit can also discuss potential participation (they have demonstrated in previous research their understanding of culturally sensitive recruitment). Adequate time will be provided to allow patients to consider whether they want to participate in the trial. Aboriginal and/or Torres Strait Islander patients can also discuss their potential participation with the Indigenous Health Liaison Officer for the Renal Service; all potential participants can discuss their participation with family/carers/friends as they wish.

A Participant Information Sheet and Consent Form will be provided to all potential participants. These documents can be read to potential participants if required. Written consent will be obtained by the patient signing with their name or an X. Participants’ consent will be requested for the collection of demographic characteristics such as: age; ethnicity; living arrangements (whether it is shared accommodation (hostel) or private house; social supports; whether the patient has had to relocate to Townsville for dialysis; distance travelled each day for dialysis); length of time since commenced on dialysis; duration of each dialysis session; intradialytic weight gain and attendance records. Previous research conducted in the renal unit by this team has been successful in terms of participant recruitment [for example, 19].

##### 2.9.2.2 Recruitment of clinician participants

All clinical staff in the unit will be invited to participate. They will be informed of the study by way of flyers/ward meetings/ward communication book, and email including a Quick Response (QR) code to the on-line questionnaire. Information will be provided to clinical staff by way of Participant Information Sheet; consent will be provided by checking a box that states, “I agree to complete this questionnaire”, and on-line survey logic will be applied.

#### 2.9.3 Withdrawal of participants from the project

There are no consequences for declining to participate in this trial or withdrawing from the study. On any given day during the intervention phase, participants can choose not to wear the headset. Data collected prior to withdrawal will be retained and included in the analysis on an intention-to-treat basis.

#### 2.9.4 Randomization

**Fig. 1:**
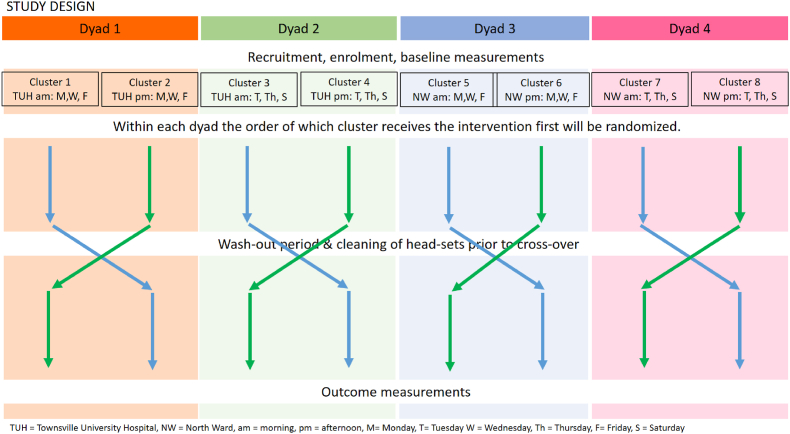
Design of Cross-over Randomised Controlled feasibility study.

There will be 8 clusters of patients to be randomised (4 × 2 dyads) organised by location of dialysis (TUH or NW), day of the week (Monday-Wednesday-Friday roster or Tuesday-Thursday-Saturday roster) and time of dialysis (morning or afternoon). Randomization will be to VR (intervention) or usual activities (control) (Fig. 1).

To avoid contamination, clusters will be randomised (using web-based computer program, for example www.randomization.com) [27] within dyads as to which cluster receives VR first. Whilst it is not possible to ‘blind’ participants and unit staff to the allocation, the investigators analysing the data will be blinded to group assignment.

#### 2.9.5 Data collection processes

So as not to inconvenience patient participants' routines, the conversational interview about their experiences with VR, and the data related to quality of life and psychological measures will take place during a scheduled dialysis session (in the week following the 4-week intervention/control periods). All data collected directly from the participants will be undertaken in a conversational manner in their familiar surroundings. If the patient prefers, data collection can be undertaken in an office in the renal unit; it is recognised, however, that this would be a less inviting environment and may inhibit conversation. Measures to be used are described earlier in sections 2.9.1.2, 2.9.1.3, and 2.9.1.4. After recruitment and consent, but before their first haemodialysis session associated with the trial, participants will be asked to complete the AQoL-6D and the K-5 at three timepoints: the week prior to first haemodialysis session associated with the trial, the one-week ‘washout period’ between the intervention and control phase (or vice versa) and in the week following involvement in the trial. (Refer to Fig. 1). During the intervention phase, the research assistant/Health Workers/nurses will seek formative feedback about the use of the VR headset. The summative interview (based on the User Engagement scale) will be conducted in the week following completion of the intervention period.

Socio-demographic and clinical related characteristics of participants such as age, gender, relationship status, place of residence, age at initiation of haemodialysis, type of vascular access, cause of renal failure) will be obtained from the participants' medical records by the study's Research Assistant, after patient consent has been obtained. Attendance and intradialytic weight gain will be retrieved from the electronical medical record for each dialysis session.

##### 2.9.5.1 Data collection/gathering techniques and information to be gathered relating to conversational semi-structured interview with patients

Conversational semi-structured interviews will be conducted with the patient participants. The Clinical Nurse Consultant – Indigenous at TUH, has reviewed and approved the questions on the interview schedule as being culturally and linguistically appropriate. The Health Workers – Renal and Research Assistants will be provided training/practice to undertake the conversational interviews. To allow flexibility and spontaneity during the interviews, interview questions may not be asked in the same order or wording as in the interview guide. However, all questions in the interview guide will be discussed during each interview. Questions relating to any difficulties encountered while using the VR headset, experience of any kind of motion sickness will be included.

##### 2.9.5.2 Administration of surveys/questionnaires to patients

Four standardised questionnaires will be administered to the participants. The measures are detailed in sections 2.9.1.2, 2.9.1.3, 2.9.1.4. A conversational approach will be used to elicit information from all participants during the administration of the questionnaires.

##### 2.9.5.3 Administration of surveys/questionnaires to clinical staff

Clinical staff will be asked to complete an anonymous questionnaire, adapted from the amended Lindner et al. (2019), satisfaction scale [26] after all dyads have completed the trial. The survey will be accessed via a QR code or a web address.

#### 2.9.6 Data management and storage

Patients participating in the study will have a unique study number assigned to them. Data pertaining to conversational interviews with the patient participant group will have identifiers removed following analysis. Data pertaining to attendance, intradialytic weight gain, and psychological measures will be re-identifiable at collection; identifiers will be removed following data cleaning and analysis. Anonymous survey data from the clinicians will be collected electronically through the survey tool hosted on the Qualtrics platform. Partially completed survey responses will be included. The Qualtrics survey data will be hosted on the James Cook University server. Other study documents will be stored at TUH.

All data obtained will remain confidential and will be securely stored throughout the course of the study within locked cupboards/filing cabinets, to which only the research team members have access. Computer files will be accessible only to the research team. Participants in the trial will not be identifiable in any reports, publications or presentations arising from the study. This study has ethics approval from the Health service's Human Research and Ethics Committee (HREC/QTHS/72273).

#### 2.9.7 Data analysis plan

The quantitative data will be entered into an IBM SPSS software (SPSS) database (Version 25, Chicago). Chi-square tests will be used to analyse categorical data. Ordinal data will be analysed via rank tests. Paired t-tests and ANOVA will be used for continuous variables where appropriate. Where assumptions of normality have been violated, non-parametric versions of tests will be used. If participants withdraw, attendance data will be analysed by intention-to-treat. Where appropriate, modelling of outcomes will be undertaken in order to explain variability in results.

Qualitative analysis will include notes taken from informal interviews with patients and will be analysed deductively. Observational and summary notes taken by the investigators, and notes taken by a Stakeholder Group (comprising patient, staff and researcher representatives, who will meet to discuss the progress of the study at least monthly) will also form part of the analysis.

#### 2.9.8 Safety considerations/Patient safety

The headsets will be able to be removed at any time, for example if the participant feels nauseous or unwell. Each headset will be allocated to a single patient for their use while they are in the intervention arm of the trial. After each use, the headset will be wiped with a detergent solution or wipe. The headsets will be sanitised according to infection prevention and control procedures at TUH prior to allocation to another participant. The study design allows for a week between intervention and control periods for this to occur. The ‘Virtual reality head mounted display cleaning and disinfection guide’ produced by the Health Service's Clinical Education and Simulation Services, and any specific manufacturer's instructions will be followed with respect to cleaning the headsets, thereby preventing spread of infection between users.

The safety of the patients participating in this study has been considered in the design of the RCT. We will use immersive VR headsets that do not require tethering to a computer. They will only be used when the participant is seated in the chair during their dialysis session so there will no increased risk of falling while using the headsets. From the recently conducted systematic review about VR in haemodialysis [6], it is safe for patients to access VR at any time during their dialysis – beginning, middle, or toward the end. All patients in the trial will be monitored by the nurse allocated to their care, as per usual practice. Nurses will assist patients to put on the headsets, adjust them, and access the virtual reality experiences. Nurses will also be present to remove the headset for any reason. The headsets can be quickly removed in the case of a medical emergency. Any emerging safety concerns will be brought to the attention of the Stakeholder Group, and the treating clinician as appropriate.

## 3 Discussion

This study design has been informed by a systematic literature review by team members on the impact of VR experiences for patients undergoing haemodialysis. The recent significant media coverage of our research indicates the public's interest in this topic and has resulted in knowledge transfer on the topic.

This study will ascertain acceptability and feasibility of offering an immersive VR experience to patients attending haemodialysis in a busy clinical setting. Results from this crossover study will be used to inform a multi-centre RCT that will test the effectiveness of VR on the lives of people receiving haemodialysis across northern Australia. Improvements in the engagement of individuals with their care is known to increase self-efficacy and general sense of wellbeing. This study highlights the importance of evaluating interventions that aim to make clinical environments more acceptable to persons who have ongoing need for healthcare interventions.

## Declaration of interests

The authors declare that they have no known competing financial interests or personal relationships that could have appeared to influence the work reported in this paper.

## References

[1] Som A. (2017). Improving dialysis adherence for high risk patients using automated messaging: proof of concept. Sci. Rep..

[2] Smyth W., Hartig V., Hayes M., Manickam V. (2015). Patients' adherence to aspects of haemodialysis regimens in tropical North Queensland, Australia. J. Ren. Care.

[3] Pancras G., Shayo J., Anaeli A. (2018). Non-medical facilitators and barriers towards accessing haemodialysis services: an exploration of ethical challenges. BMC Nephrol..

[4] Reilly R. (2016). Effectiveness, cost effectiveness, acceptability and implementation barriers/enablers of chronic kidney disease management programs for Indigenous people in Australia, New Zealand and Canada: a systematic review of mixed evidence. BMC Health Serv. Res..

[5] (2019). Townsville Hospital and Health Service, *2018-2019 Annual Report*.

[6] Omonaiye O., Smyth W., Nagle C. (2021). Impact of virtual reality interventions on haemodialysis patients: a scoping review. (published on-line 24 January 2021). J. Ren. Care.

[7] Marín-Morales J. (2019). Real vs. immersive-virtual emotional experience: analysis of psycho-physiological patterns in a free exploration of an art museum. PLoS One.

[8] Roettl J., Terlutter R. (2018). The same video game in 2D, 3D or virtual reality - how does technology impact game evaluation and brand placements?. PLoS One.

[9] Chirico A., Cipresso P., Yaden D.B., Biassoni F., Riva G., Gaggioli A. (Apr 27 2017). Effectiveness of immersive videos in inducing awe: an experimental study. Sci. Rep..

[10] Quesnel D., Riecke B.E. (2018). Are you awed yet? How virtual reality gives us awe and goose bumps. Front. Psychol..

[11] Ventura S., Brivio E., Riva G., Baños R.M. (2019). Immersive versus non-immersive experience: exploring the feasibility of memory assessment through 360° technology. Front. Psychol..

[12] Zhou H. (2020). Application of wearables to facilitate virtually supervised intradialytic exercise for reducing depression symptoms. Sensors (Basel, Switzerland).

[13] Maynard L.G. (2019). Effects of exercise training combined with virtual reality in functionality and health-related quality of life of patients on hemodialysis. Game. Health J..

[14] Solimini A.G. (2013). Are there side effects to watching 3D movies? A prospective crossover observational study on visually induced motion sickness. PLoS One.

[15] Burrows B., Wilund K., Hernandez R. (2020). Fully immersive virtual reality for hemodialysis patients: is it safe?. Blood Purif. Conf. Abstract.

[16] Polit D., Beck C.T. (2017).

[17] Chang E., Kim H.T., Yoo B. (2020/10/20 2020). Virtual reality sickness: a review of causes and measurements. Int. J. Hum. Comput. Interact..

[18] Burrai F. (2019). Effects of virtual reality in patients undergoing dialysis: study protocol. Holist. Nurs. Pract..

[19] McArdle J., Smyth W., Wicking K., Gardner A. (2017). Haemodialysis central venous catheter exit site dressings in the tropics: a crossover randomized controlled trial. Wound Pract. Res..

[20] Richardson J.R.J., Peacock S.J., Hawthorne G., Iezzi A., Elsworth G., Day N.A. (2012). Construction of the descriptive system for the assessment of quality of life AQoL-6D utility instrument. Health Qual. Life Outcome.

[21] Hawthorne G., Korn S., Richardson J. (2013). Population norms for the AQoL derived from the 2007 Australian national survey of mental health and wellbeing. Aust. N. Z. J. Publ. Health.

[22] AIHW (2009).

[23] Brinckley M.-M. (2019). https://openresearch-repository.anu.edu.au/bitstream/1885/224438/1/Brinckley%20Thesis%20Final%20Submitted.pdf.

[24] O'Brien H.L., Cairns P., Hall M. (2018). A practical approach to measuring user engagement with the refined user engagement scale (UES) and the new UES short form. Int. J. Hum. Comput. Stud..

[25] Bessarab D., Ng’andu B. (2010). Yarning about yarning as a legitimate method in Indigenous research. Int. J. Crit. Indig. Stud..

[26] Lindner P., Miloff A., Zetterlund E., Reuterskiöld L., Andersson G., Carlbring P. (2019). Attitudes towards and familiarity with virtual reality therapy among practicing cognitive behavior therapists: a cross-sectional survey study in the era of consumer VR platforms. Front. Psychol..

[27] Gerard E. Randomization.com. http://www.jerrydallal.com/random/randomize.htm.

